# Supplementary material for: Early-life immunological and microbial differences between East African and North European children
Source: Commun Med (Lond). 2026 Mar 3;6:216. doi: 10.1038/s43856-026-01482-0 (PMC13076672; doi:10.1038/s43856-026-01482-0)
Supplement: Supplementary file 2 — Supplementary Information [file 43856_2026_1482_MOESM2_ESM.pdf]

SUPPLEMENTARY INFORMATION

Early-life immunological and microbial differences between East African and North European children

Noora Nurminen[1], Yue-Mei Fan[1], Emma Kortekangas[1], Jake Lin[1], Lotta Hallamaa[1], Kenneth Maleta[2], Kirsi-Maarit Lehto[1,3], Olli H. Laitinen[1], Aki Sinkkonen[4], Johanna Lempainen[5,6,7], Jorma Toppari[6,8,9,10], Riitta Veijola[11], Kalle Kurppa[1,13,14], Mikael Knip[13,15], Ulla Ashorn[1], Sami Oikarinen[1], Per Ashorn[1], Heikki Hyöty[1,16*]

[1]Faculty of Medicine and Health Technology, Tampere University, Tampere, Finland.
[2]School of Global and Public Health, Kamuzu University of Health Sciences, Blantyre, Malawi
[3]University of Helsinki, Faculty of Veterinary Medicine, Helsinki, Finland.
[4]Natural Resources Institute Finland, Luke, Turku, Finland
[5]Immunogenetics Laboratory, Institute of Biomedicine, University of Turku, Turku, Finland
[6]Departments of Pediatrics, University of Turku and Turku University Hospital, Turku, Finland
[7]Clinical Microbiology, Turku University Hospital, Turku, Finland
[8]Centre for Population Health Research, University of Turku and Turku University Hospital, Turku, Finland.
[9]Research Centre for Integrative Physiology and Pharmacology, Institute of Biomedicine, University of Turku, Turku, Finland
[10]InFLAMES Research Flagship Centre, University of Turku and Åbo Akademi University, Turku, Finland
[11]Department of Pediatrics, Research Unit of Clinical Medicine, Medical Research Centre, Oulu University Hospital and University of Oulu, Oulu, Finland.
[13]Department of Pediatrics, Tampere University Hospital, Tampere, Finland.
[14]Seinäjoki University Consortium, Seinäjoki, Finland
[15]Research Program for Clinical and Molecular Metabolism, Faculty of Medicine, University of Helsinki, Helsinki, Finland.
[16]Fimlab Laboratories, Pirkanmaa Hospital District, Tampere, Finland.

*Corresponding Author:

Professor Heikki Hyöty
Tampere University, Faculty of Medicine and Health Technology
Arvo Ylpön katu 34, FI-33520 Tampere, Finland
Tel.: +358 505 168 480, Email: heikki.hyoty@tuni.fi

Supplementary Table 1. RT-qPCR-primers and probes.

Microbe	Name	Sequence	Conc. (μM)
**Adenovirus**	346ADVso	CCG GCC GAG AAG GGC GTG CGC AGG TA	900
	372ADVso	CAT GAC TTT TGA GGT GGA YC	300
	ADVso Probe	FAM-ATG GAT GAG CCC ACC CT-MGB	200
**Enterovirus**	fwd 636	CGGCCCCTGAATGCGGCTAA	900
	rev 4-	GAAACACGGACACCCAAAGTA	900
	Q-PCREVI	FAM-TCTGTGGCGGAACCGACTA-TAMRA	300
	Q-PCREVII	FAM-TCTGCAGCGGAACCGACTA-TAMRA	300
**Rhinovirus**	fwd 636	CGGCCCCTGAATGCGGCTAA	900
	rev 4-	GAAACACGGACACCCAAAGTA	900
	Rhinoprobe	VIC- CGGGAIGGGACCAACTA -TAMRA	300
**Norovirus**	NoroG2 fwdQ	CARGARBCNATGTTYAGRTGGATGAG	900
	NoroG2 revQ	TCGACGCCATCTTCATTCACA	300
	NoroG1 revQ:	CTTAGACGCCATCATCATTYAC	900
	NoroG1 fwdQ	CGYTGGATGCGNTTYCATGA	900
	NoroG2probe	FAM-TGG GAG GGC GAT CGC AAT CT-TAMRA	300
	NoroG1probe Q	VIC-AGA TYG CGA TCY CCT GTC CA- TAMRA	250
**Rotavirus**	VP2-F1	TCTGCAGACAGTTGAACCTATTAA	900
	VP2-F2	CAGACACGGTTGAACCCATTAA	900
	VP2-F3	TCGGCTGATACAGTAGAACCTATAAATG	900
	VP2-F4	TGTCAGCTGATACAGTAGAACCTATAAATG	900
	VP2-F5	TCAGCTGACACAGTAGAACCTATA AATG	900
	VP2-R1	GTTGGCGTTTACAGTTCGTTCAT	50
	VP2-R2	GTTGGCGTCTACAATTCGTTCAT	50
	RotaVp2-P	FAM-ATG CGC ATR TTR TCA AAH GCA A-MGB-NFQ	200
**Parechovirus**	ParE AN345	GTAACASWWGCCTCTGGGSCCAAAAG	300
	ParE AN344	GGCCCCWGRTCAGATCCAYAGT	300
	ParE AN257	FAM-CCTRYGGGTACCTYCWGGGCATCCTTC-TAMRA	200
**Enterovirus**	FW	GCCCCTGAATGCGGCTAAT	0.10 mM
	Rev	GATGGCCAATCCAATAGCT	0.10 mM
	Rev	ATTGTCACCATAAGCAGCCA	0.10 mM
**Giardia lamblia**	FW	GAC GGC TCA GGA CAA CGG TT	0.70
	Rev	TTG CCA GCG GTG TCC G	0.70
	probe	FAM-CCC GCG GCG GTC CCT GCT AG-MGB	0.10
**Cryptosporidium spp.**	FW	CTT TTT ACC AAT CAC AGA ATC ATC AGA	0.40
	Rev	TGT GTT TGC CAA TGC ATA TGA A	0.40
	probe	VIC-TCG ACT GGT ATC CCT ATA A-MGB	0.10

Supplementary Table 2. **Linear mixed-effects models for plasma cytokine concentrations.** LMM models cover samples of 6, 18 and 30 months-of-age for Malawian children and 6, 18, 24 and 36 months-of-age for Finnish children with the following covariates: country, age, breastfeeding and WAZ. Interaction between age and country is also included. Cytokine concentrations were log2 transformed for analysis.

Parameter	Estimate	Std.Error	95% CI (lower,upper)		p-value
**IL-10**					
(Intercept)	3.812	0.350	3.136	4.491	< 2e-16
Country (Malawi)	1.219	0.490	0.259	2.166	0.014
Age	-0.059	0.015	-0.088	-0.031	9.250E-05
Breastfeeding	-1.236	0.355	-1.925	-0.538	6.450E-04
WAZ	0.015	0.089	-0.157	0.188	0.863
Country:Age	0.067	0.028	0.013	0.122	0.018
**IL-6**					
(Intercept)	-1.963	0.417	-2.776	-1.155	5.270E-06
Country (Malawi)	5.227	0.581	4.096	6.353	5.160E-16
Age	0.096	0.017	0.064	0.129	5.320E-08
Breastfeeding	-1.346	0.416	-2.159	-0.507	0.001
WAZ	-0.015	0.114	-0.235	0.206	0.897
Country:Age	-0.174	0.032	-0.236	-0.112	2.280E-07
**IL-1b**					
(Intercept)	-0.150	0.254	-0.642	0.344	0.557
Country (Malawi)	1.591	0.353	0.895	2.277	1.250E-05
Age	-0.006	0.011	-0.027	0.015	0.588
Breastfeeding	-0.966	0.258	-1.464	-0.461	2.460E-04
WAZ	-0.072	0.066	-0.200	0.056	0.277
Country:Age	-0.007	0.020	-0.045	0.033	0.734
**TNF-a**					
(Intercept)	3.847	0.216	3.429	4.265	< 2e-16
Country (Malawi)	0.306	0.303	-0.281	0.893	0.314
Age	-0.065	0.009	-0.084	-0.047	5.470E-11
Breastfeeding	-0.107	0.219	-0.532	0.318	0.626
WAZ	-0.083	0.051	-0.182	0.016	0.107
Country:Age	0.037	0.018	0.003	0.072	0.039

Supplementary Table 3. **Linear mixed-effects model for gut microbial alpha diversity.** The LMM model covers stool samples of 6, 18 and 30 months with the following covariates: country, age, breastfeeding and WAZ. Interaction between age and country is also included.

Parameter	Estimate	Std.Error	95% CI (lower,upper)		p-value
(Intercept)	0.736	0.034	0.670	0.802	< 2e-16
Country (Malawi)	-0.050	0.044	-0.135	0.035	0.256
Age	0.006	0.002	0.003	0.009	0.000542
Breastfeeding	-0.035	0.033	-0.098	0.028	0.284
WAZ	-0.004	0.008	-0.019	0.011	0.617
Country:Age	0.001	0.003	-0.005	0.006	0.808

Supplementary Table 4. **Daily consumption of different food categories in Malawian and Finnish children.** The data is derived from seven-day food frequency questionnaire data for Malawian children at 18 months-of-age whereas data for the Finnish children was derived from Kyttälä et al. 2010 three-day food record data in the same DIPP study that the samples are collected in this study at 12 and 24 months-of-age[1]. Percent of daily consumers was calculated for each food category in order to harmonize the data. An average % of daily consumers was calculated from 12 months and 24 months-of-age for the Finnish children since the data did not have food record data at 18 months-of-age.

**Food Category**	**Daily consumers (%)**	
	**Malawi 18 m**	**Finland 12 + 24 m average**
Cereals	97 %	100 %
Fruit/berries	28 %	95 %
Vegetables	64 %	73 %
Meat	0 %	100 %
Fish	3 %	42 %
Dairy	8 %	94 %
Fat	46 %	73 %

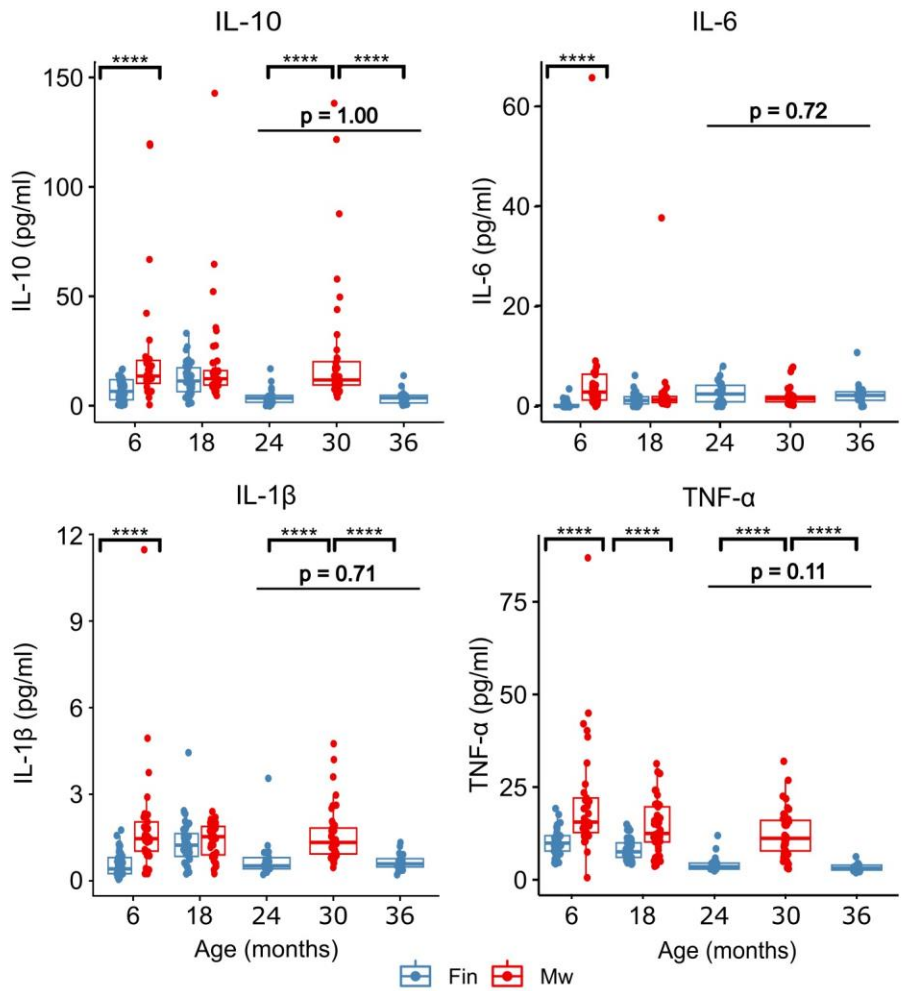

Supplementary Figure S1. Plasma cytokine levels in Finnish and Malawian children in all time points. P-values for two-sided Mann-Whitney U test comparing Finnish 24-month and 36-month samples are shown separately, otherwise **** $P \leq 0.0001$ between the groups.

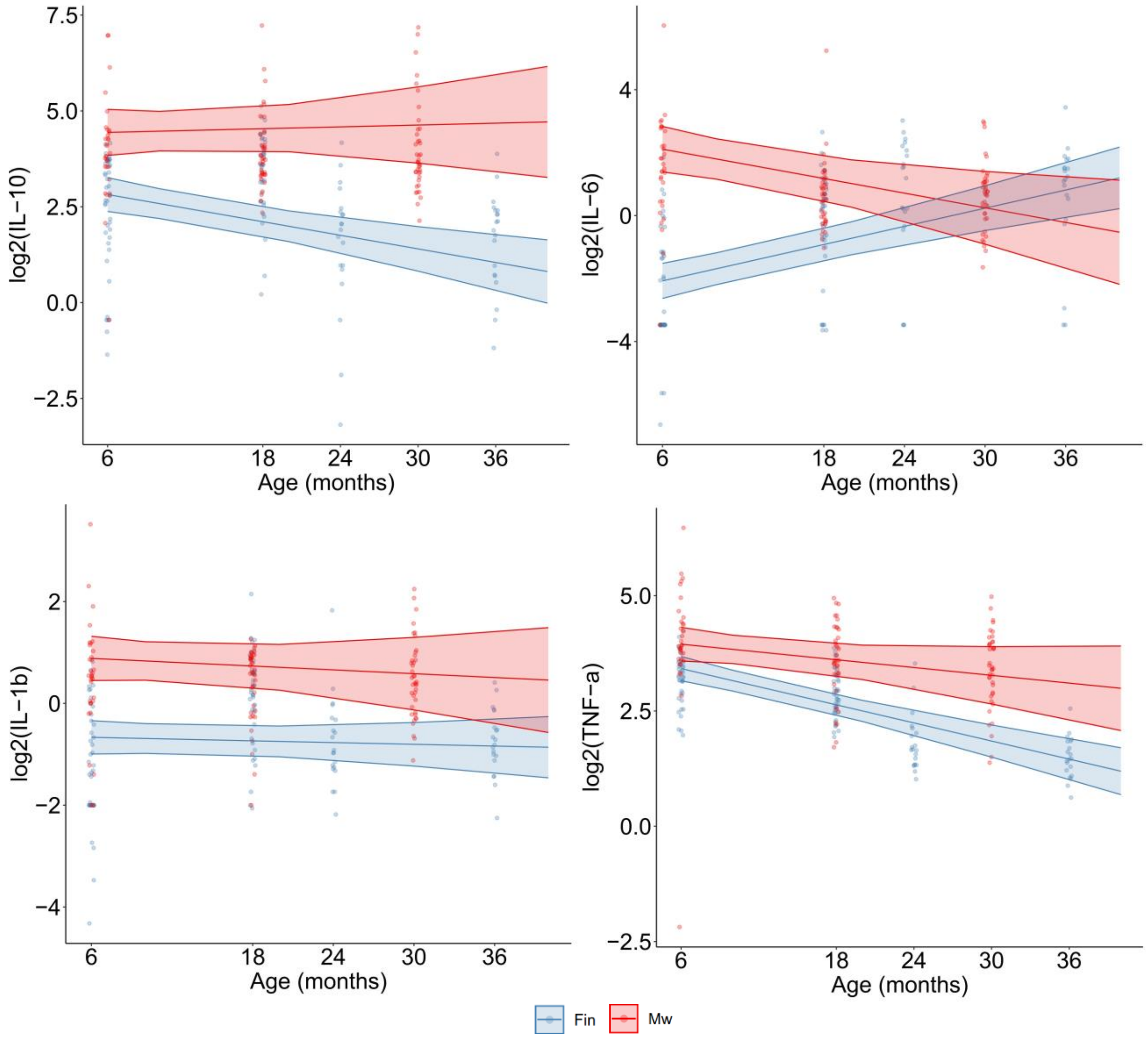

Supplementary Figure S2. Modification of the association between age and plasma cytokines by country using linear mixed-effects models. The models cover samples of 6, 18, 24 and 36 months-of-age for Finnish children, and 6, 18 and 30 months-of-age for Malawian children with the following covariates: country, age, breastfeeding and WAZ and interaction between age and country. Cytokine concentrations were log2 transformed for analysis.

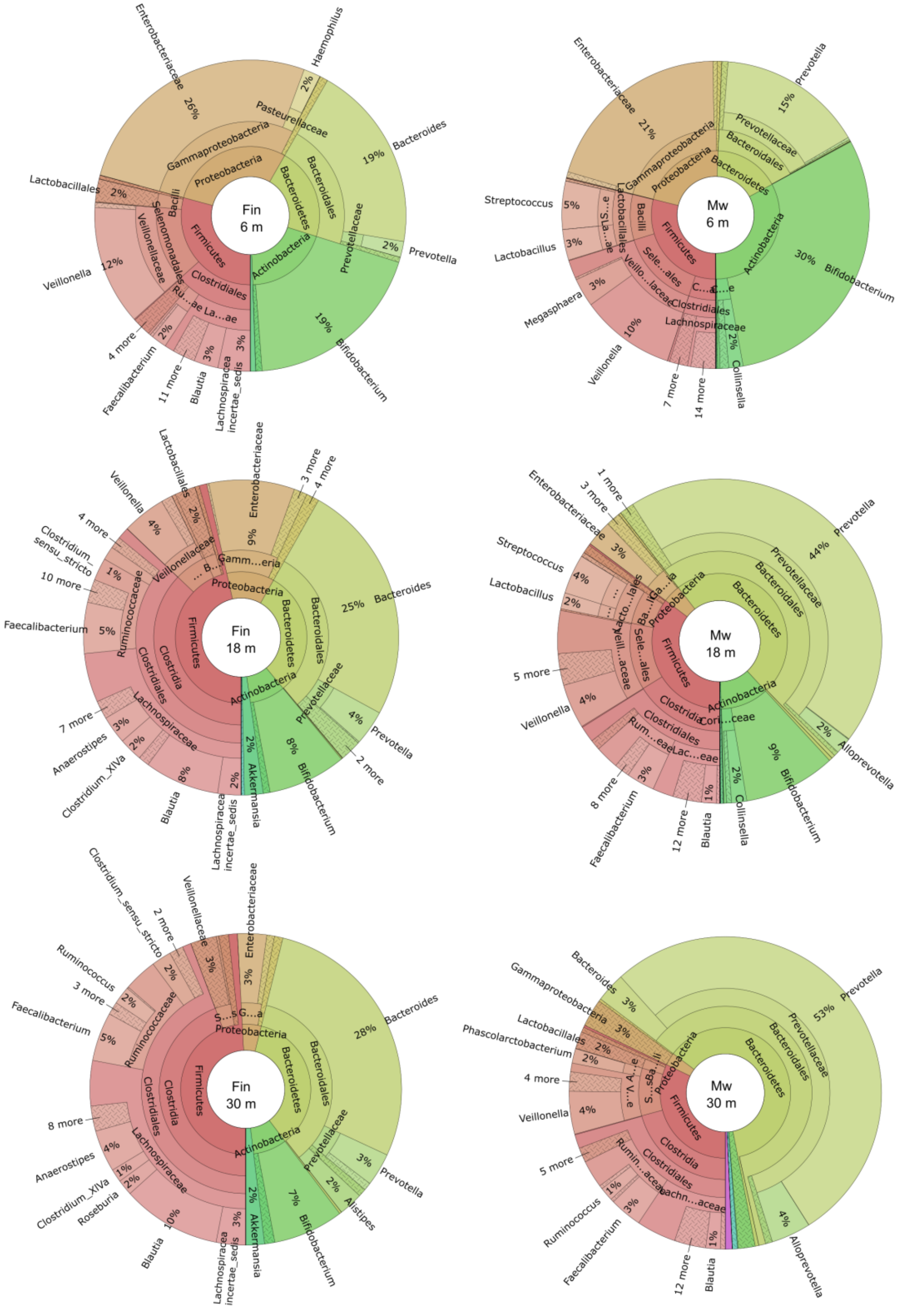

Supplementary Figure S3. The fecal taxa composition of Finnish and Malawian children at 6, 18, and 30 months of age. Figure was produced with Krona[2]. The Krona plots reveal a process of microbiota maturation into *Bacteroides*-dominating microbiota in Finnish children, and *Prevotella*-dominating microbiota in Malawian children.

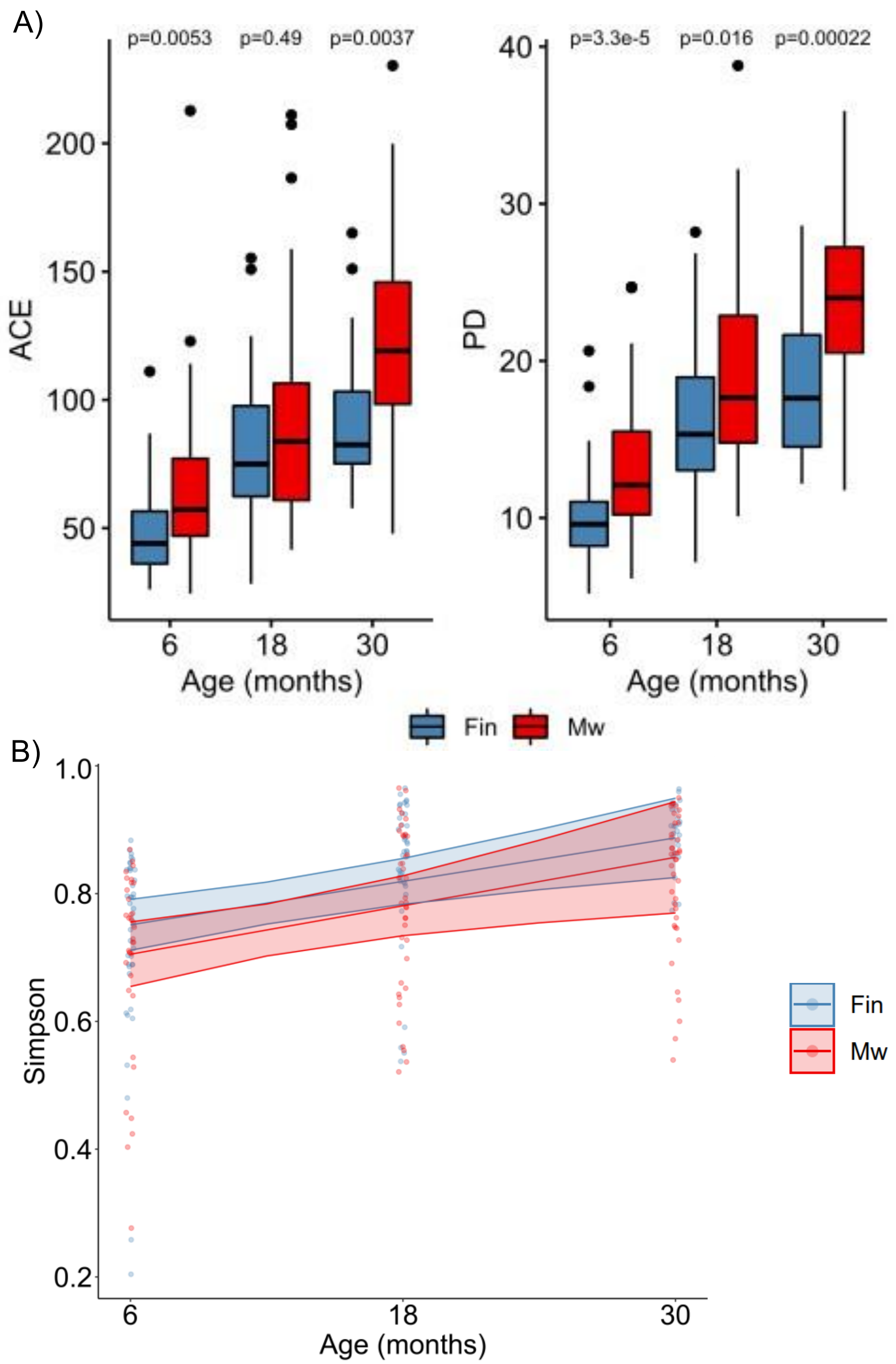

Supplementary Figure S4. The alpha diversity of gut microbiota in Finnish and Malawian children at 6, 18, and 30 months of age. A) Alpha diversity measured as Abundance-based Coverage Estimator (ACE) and Faith's Phylogenetic diversity (PD) index. The medians with interquartile ranges are indicated and differences are analyzed using two-sided Mann-Whitney U test. **B)** Linear mixed-effects model for Simpson diversity. The model includes country, age, breastfeeding and WAZ as covariates. Interaction between age and country is also included.

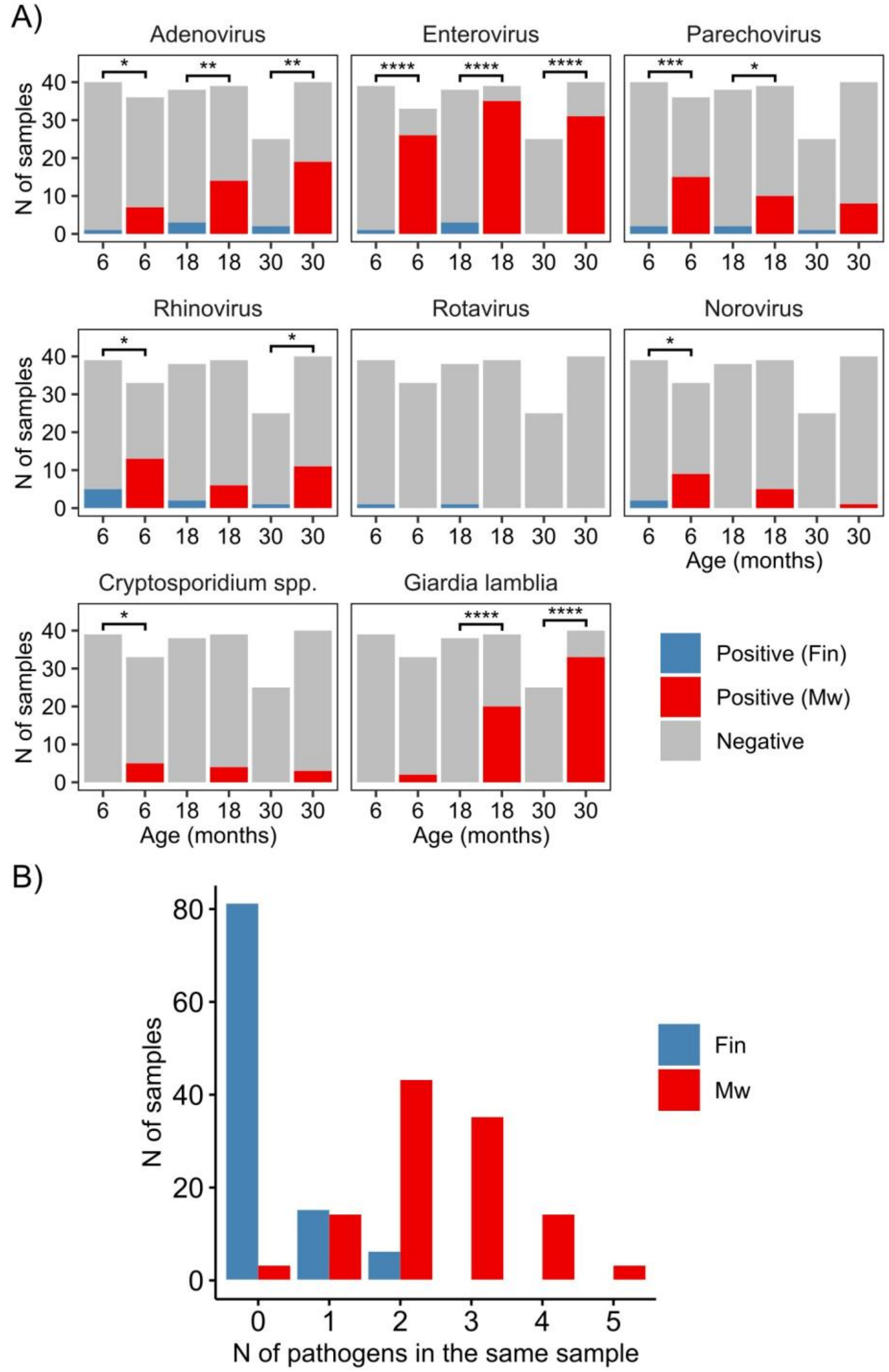

Supplementary Figure S5. Pathogen positivity and number of detected pathogens in Finnish and Malawian stool samples presented as number of samples. The pathogens were detected in stool samples taken at 6, 18, and 30 months of age using RT-qPCR. **A)** Number of positive samples presented in each age group. Differences between the countries are analyzed using Fisher's exact test. $\star$ $P < 0.05$, $\star\star$ $P < 0.01$, $\star\star\star$ $P < 0.001$, $\star\star\star\star$ $P < 0.0001$ between the groups. **B)** Number of detected pathogens in stool samples of Finnish and Malawian children. Malawian children had more often multiple pathogen positivity in the same sample than Finnish children (84.8% vs. 5.9%, P < 0.001 Fisher's exact test).

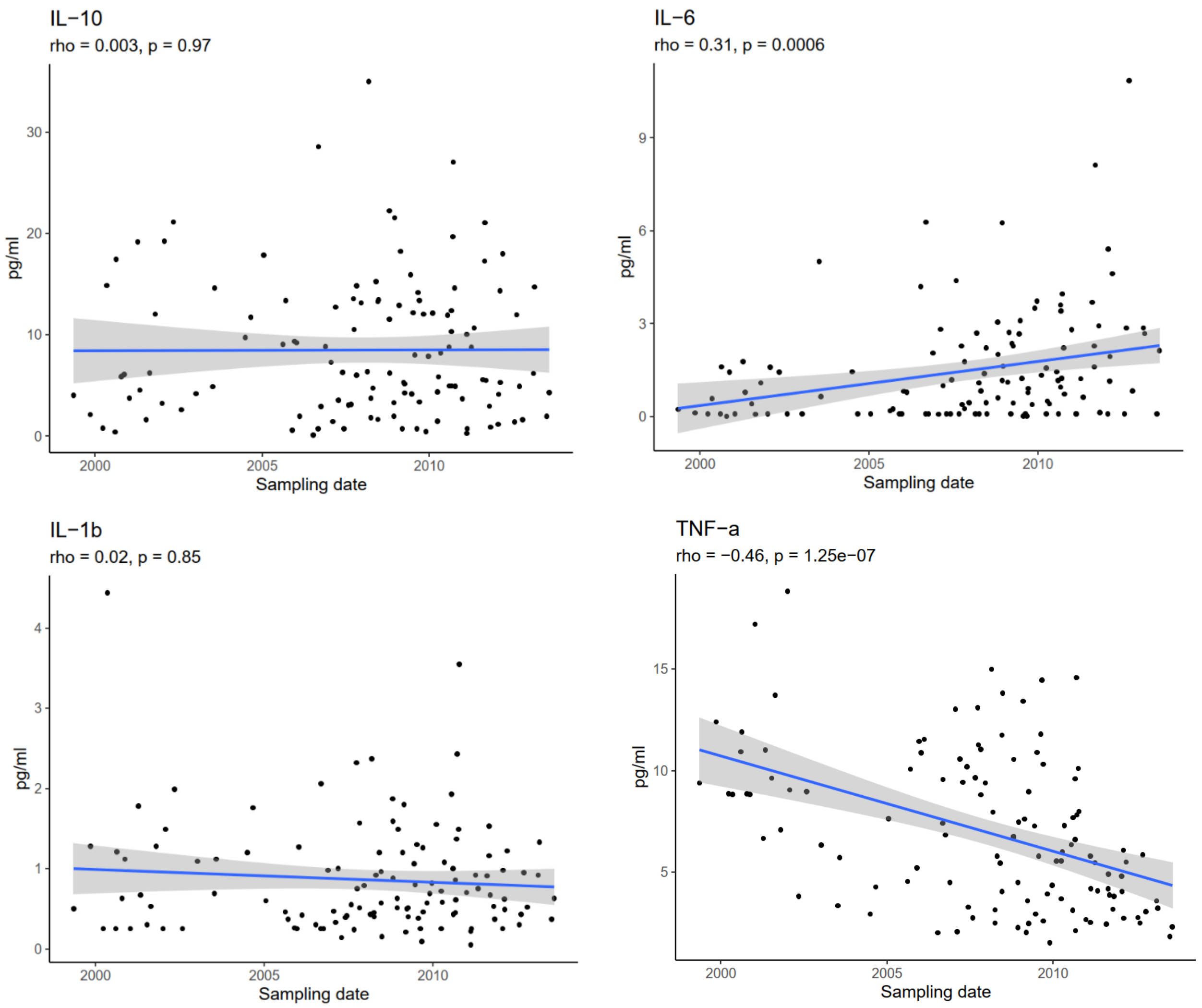

Supplementary Figure S6. Finnish plasma cytokine levels against sampling date. The Concentration of IL-10, IL-6, IL-1b, and TNF-a are plotted against sampling date. Correlations are analyzed using Spearman's rank-order correlation.

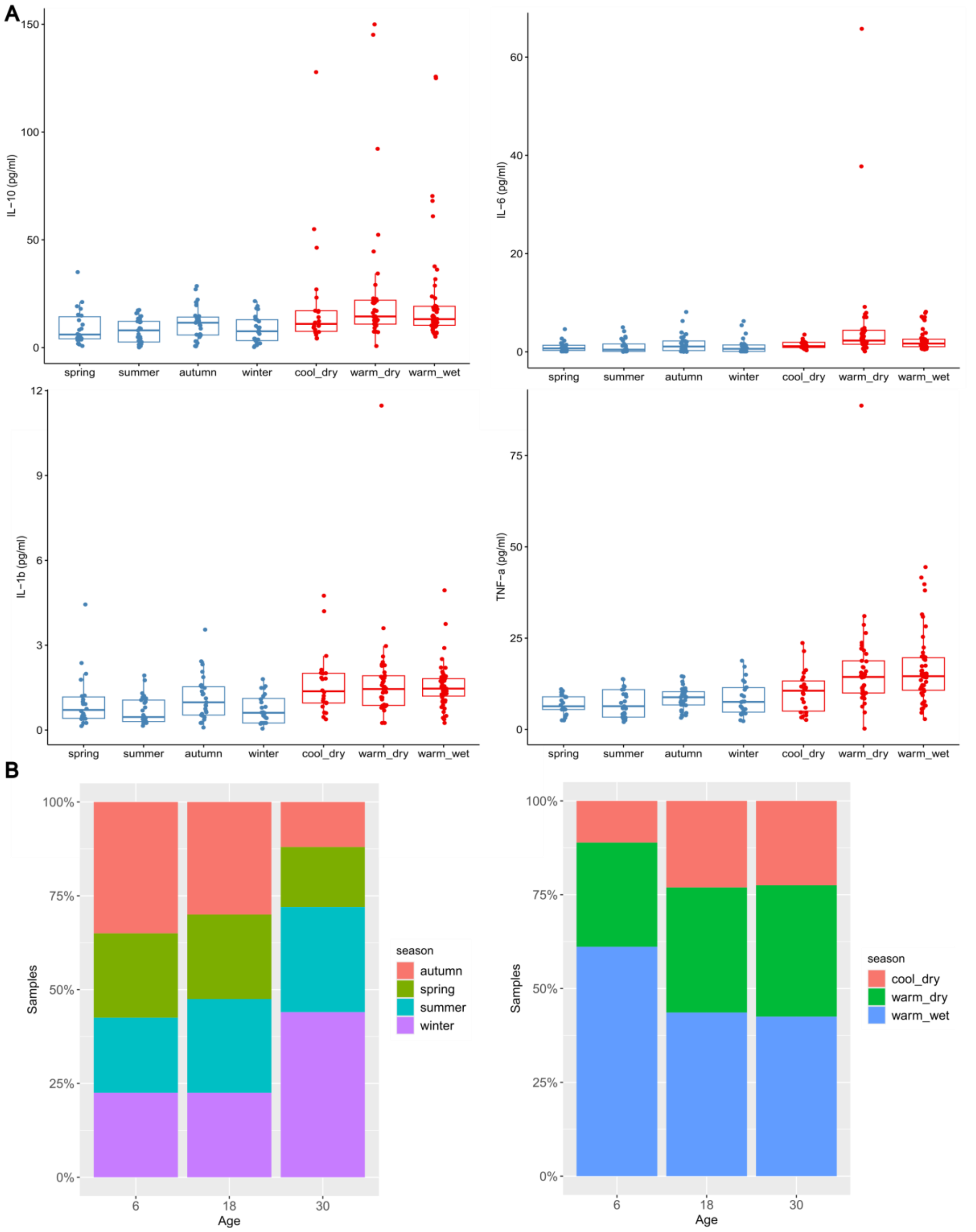

Supplementary Figure S7. Cytokine concentrations in different seasons in Finnish and Malawian plasma samples and sampling season distribution. A) Plasma IL-10, IL-6, IL-1b, and TNF-a concentration in different sampling seasons in Finnish (blue) and Malawian (red) children. There are four seasons in Finland and three seasons in Malawi. **B)** Distribution of sampling season at different sampling ages in Finnish (left) and Malawian (right) samples.

References

1. Kyttälä, P. *et al.* Food consumption and nutrient intake in Finnish 1–6-year-old children. *Public Health Nutr.* **13**, 947–956 (2010).
2. Ondov, B. D., Bergman, N. H. & Phillippy, A. M. Interactive metagenomic visualization in a Web browser. *BMC Bioinformatics* **12**, 385 (2011).
